# Solvent Front Position Extraction and some conventional sample preparation techniques for the determination of coccidiostats in poultry feed by LC–MS/MS

**DOI:** 10.1038/s41598-022-07587-5

**Published:** 2022-03-08

**Authors:** Maciej Jan Rybicki, Ilse Becue, Els Daeseleire, Anna Klimek-Turek, Tadeusz Henryk Dzido

**Affiliations:** 1 Department of Physical Chemistry, Medical University of Lublin, ul. Chodźki 4a, 20-093 Lublin, Poland; 2 Flanders Research Institute for Agriculture, Fisheries and Food, Technology and Food Science Unit (ILVO), Brusselsesteenweg 370, 9090 Melle, Belgium

**Keywords:** Analytical chemistry, Physical chemistry

## Abstract

Solvent Front Position Extraction is a novel technique developed for effective sample preparation of biological samples containing coccidiostats prior to LC–MS/MS. In this study the technique was used for isolation and determination of seven coccidiostats, from both main groups being: ionophores and chemical coccidiostats. Its effectiveness was evaluated by comparing with other sample preparation procedures, used in European routine laboratories. Results obtained by Solvent Front Position Extraction were very satisfactory (linearity R^2^ ≥ 0.971, recovery 90.1–111.1%, RSDr: 8.7–16.6%, RSD_R_: 9.0–17.7%) and fulfilled requirements described in Commission Regulation (EU) 2021/808 of 22 March 2021, which showed great potential of the technique in sample preparation of coccidiostats in poultry feed.

## Introduction

In recent years there is a clear trend towards reducing the amount of medications added to animal feed for therapeutic and preventive purposes^1,2^, except the coccidiostats, which fight against coccidiosis, a serious parasitic disease among birds, especially poultry^3^. Statistics shows, that a percentage of animal feed containing these drugs is constantly high^1,4^, which leads to their presence in food^5^. They are registered and mainly used as Feed Additives (FAs)^6^. Some coccidiostats are harmful to human health^7,8^ and life^9,10^, so proper feed control is essential to prevent poisoning. In Regulation (EC) No 183/2005^11^ European Union legislation set requirements for feed hygiene, while in Regulation (EU) No 574/2011^12^ set highest safe concentrations of coccidiostats (Maximum Limit, ML) in fodder, which the Member States are obliged to comply with. To monitor presence of coccidiostats in samples liquid chromatography combined with mass spectrometry is mainly used^3^. In every analytical method the most critical point is sample preparation. Until now, many effective methods^13–15^ for the isolation and determination of coccidiostats have been developed, but their disadvantages were often the high cost of SPE columns or the complex and time-consuming procedures. Solvent Front Position Extraction (SFPE) can be considered as an interesting and effective alternative for them, due to the automation of the procedure. It is based on thin-layer chromatography^16^. The liquid sample is applied directly on the surface of the chromatography plate. After evaporation of the sample solvent, the mobile phase with a low elution strength is distributed by movable pipette^17,18^ and develops the chromatogram horizontally. In a second step, a second solvent is used with an elution strength high enough to allow the substance(s) of interest and internal standard to follow up with the solvent front. The chromatogram is developed in the same direction as in the first step to a distance of a few mm longer than the least-retained substance of interest. The zone of the substance of interest and the internal standard is usually visible without derivatization under white light illumination or UV lamp, so they can be easily extracted from the surface of sorbent for further LC–MS/MS analysis, hence the name Solvent Front Position Extraction. SFPE was first introduced in 2019^16^. It allowed to effectively separate 5 coccidiostats from matrix elements and determine them quantitatively. In the latest work from 2020^19^, SFPE was optimized for the development of chromatograms of pure coccidiostats with a prototype of a semiautomatic device with a moving pipette for delivering the eluent to the chromatography plate for the first time and it allowed quantification of coccidiostats in samples of two commercially available premixes, whose coccidiostats content was in the range 12–19.4%. In this work the main goal was a quantification of 7 (maduramycin, narasin, salinomycin, monensin, lasalocid, robenidine, nicarbazin and nigericin as an internal standard) coccidiostats simultaneously in feed samples at a ML concentrations, which values are much lower than before (μg/kg) due to optimization of method for practical use. ML means Maximum Level of concentration values of coccidiostats allowed in feed due to the inevitable transfer of coccidiostats from the target feed to non-target feed during manufacturing process—to protect sensitive animals. It was expected, that SFPE coupled with LC–MS/MS could be potentially used as reference method for determination of coccidiostats. For this reason SFPE was validated according to the European Commission Regulation 2021/808 of 22 March 2021^20^ parallelly with 3 other sample preparation techniques for coccidiostats^21–23^, established by or with cooperation with European routine laboratories^24,25^. Obtained validation parameters were compared and used to evaluate usability of SFPE.

## Experimental

### Substances and reagents

Coccidiostats: maduramycin ammonium, narasin, salinomycin sodium salt, monensin sodium salt, lasalocid A sodium salt, robenidine hydrochloride, dinitrocarbanilide (marker for nicarbazin) and nigericin sodium salt were purchased from Sigma-Aldrich (St. Louis, MO, USA). The feed samples were kindly provided by the National Feed Laboratory in Lublin. Anhydrous sodium sulphate and magnesium sulphate were purchased from Alfa Aesar (Thermo Fischer, GmbH, Germany). 500 mg SPE columns (6 ml volume, Bakerbond spe WP-CBX) were provided by Avantor (Gliwice, Poland). HPTLC plates Silica gel 60 F254 and 98% formic acid solution were supplied by Merck (Darmstadt, Germany). Acetonitrile and methanol (both MS purity) were purchased from Biosolve Chemie (Dieuze, France), while for SFPE experiments: acetonitrile, methanol and toluene (all MS purity) were provided by POCH (Gliwice, Poland).

### Devices and Instrumentation

The following devices and instruments were used in this research: SL 40R centrifuge (Thermo Scientific Germany), SM-30 horizontal shaker (Edmund Buhler GmbH Germany), Grant JB Nova water bath (Grant Instruments Ltd Cambridge, UK), Milli-Q Merck water purification device (Darmstadt, Germany), Eppendorf Research Plus pipette set (Eppendorf AG Hamburg, Germany), chromatographic plate cutter (CAMAG, Muttenz, Switzerland), automatic graduated pipette (Pipetman, Gilson Company, Inc., Lewis Center, OH, USA), horizontal DS chamber for thin layer chromatography on 10 cm × 10 cm chromatography plates (Chromdes, Lublin, Poland), CAMAG TLC-MS Interface device for the extraction of analyte substances from the sorbent surface, CAMAG TLC visualizer for the detection and registration of substance zones on the surface of chromatographic plates (CAMAG, Muttenz, Switzerland), CAMAG TLC Visualizer computer with WinCATS software (WinCATS-4, CAMAG, Muttenz, Switzerland), Pol-Eko 115 SLW laboratory dryer. 21 (Pol-Eko-Aparatura, Wodzisław Śląski, Poland), analytical balance WPA 60/K, class I (RAD WAG, Radom, Poland), a prototype of a semiautomatic device with a moving pipette for delivering the eluent to the chromatography plate (Department of Physical Chemistry, Lublin, Poland), Agilent 1290 Infinity LC System (Santa Clara, CA, USA) coupled to an Agilent 6460 Triple Quadrupole, Waters Acquity H-Class FTN H-PLUS (Waters Corporation, Milford, MA, USA) combined with a Waters Xevo TQ-XS.

### CAMAG TLC-MS interface device extraction conditions

The extracting mobile phase consisted of pure methanol. Flow rate was 0.4 ml/min.

### LC–MS/MS analysis conditions

For Procedure 1 (see “Procedure 1—Solvent Front Position Extraction” section) an Agilent 1290 Infinity LC System (Santa Clara, CA, USA) coupled to an Agilent 6460 Triple Quadrupole was used. Chromatography was performed using a Zorbax Eclipse Plus C18 column (4.6 × 100 mm, 3.5 µm). Isocratic elution was performed, with 95% of 0.1% formic acid in methanol (A) and 5% of 0.1% formic acid in water for 15 min. Flow rate was 0.5 ml/min. MS data were obtained in positive and negative ionization mode (multiple reaction monitoring mode) at electrospray probe voltage 3500 V. The nebulizer gas setting was 40 psi. The ion source was operated at a temperature of 300 °C and a drying gas setting of 7 L/min.

For Procedures 2, 3 and 4 (see “Procedure 2”–“Procedure 4” sections, respectively) a Waters Acquity H-Class FTN H-PLUS (Waters Corporation, Milford, MA, USA) combined with a Waters Xevo TQ-XS was used. Chromatography was performed using an ACQUITY UPLC BEH C18 column (2.1 × 100 mm, 1.7 µm). The mobile phase consisted of: solvent A: 0.05% acetic acid in water and solvent B: 0.05% acetic acid in acetonitrile with water (50:50, v/v). The gradient elution was performed as follows: 0 min: 100% A; 9.3 min: 5% A, 95% B; 12.3 min: 100% B; 15 min: 100% A. Flow rate was 0.4 ml/min. MS data were obtained in positive and negative ionization mode (multiple reaction monitoring mode) with an electrospray probe voltage of 3500 V. The nebulizer gas setting was 40 psi. The ion source was operated at a temperature of 300 °C and a drying gas setting of 7 L/min.

### Preparation of stock solutions

Stock solutions were prepared by weighing the appropriate amount of each substance and dissolving it in methanol (except for nicarbazin, which was dissolved in DMSO). The obtained solutions were stored in a refrigerator at − 20 °C. For each coccidiostat, the concentration of the stock solution was 1000 µg/ml.

### Preparation of solutions for quantification

Working solution was prepared from the stock solutions to fortify the pure feed samples to the concentrations listed in Table 1. An internal standard solution (nigericin) was added for each sample to achieve a constant concentration of 0.25 mg/kg. MLs established by European Union legislation were chosen as reference^12^.

**Table 1:** Concentrations of individual coccidiostats in the feed samples used to plot the calibration curve points listed in the table (mg/kg).

Substance	0.25 ML	0.5 ML	1 ML	1.25 ML	1.5 ML	1.75 ML	2 ML	4 ML
Robenidine	0.175	0.35	0.7	0.875	1.05	1.225	1.4	2.8
Nicarbazin (DNC)	0.125	0.25	0.5	0.625	0.75	0.875	1	2
Lasalocid	0.3125	0.625	1.25	1.5625	1.875	2.1875	2.5	5
Salinomycin	0.175	0.35	0.7	0.875	1.05	1.225	1.4	2.8
Monensin	0.3125	0.625	1.25	1.5625	1.875	2.1875	2.5	5
Narasin	0.175	0.35	0.7	0.875	1.05	1.225	1.4	2.8
Maduramycin	0.0125	0.025	0.05	0.0625	0.075	0.0875	0.1	0.2

### Extraction procedures

#### Procedure 1—Solvent Front Position Extraction

**Figure 1:**
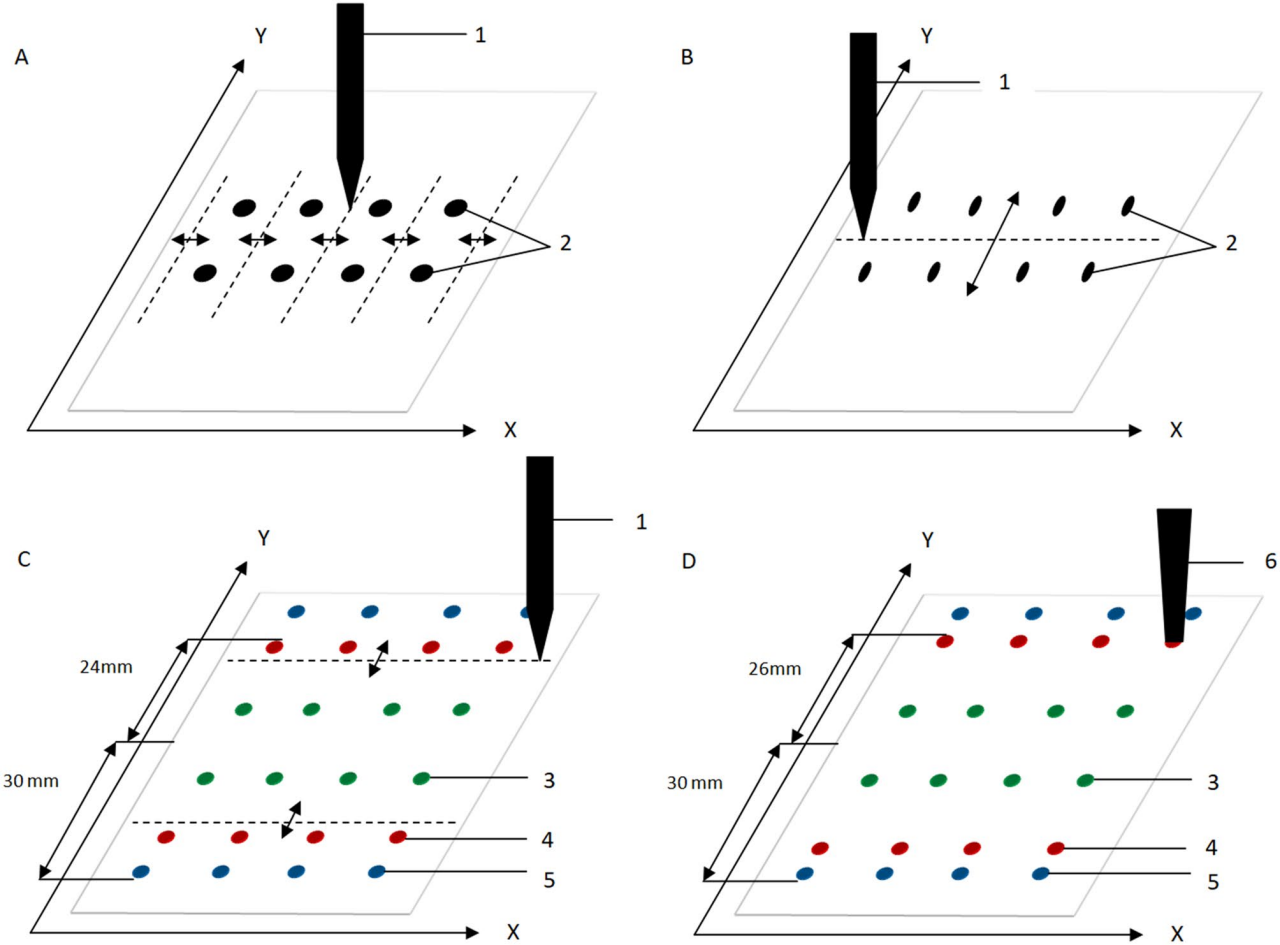
Steps of sample preparation by Solvent Front Position Extraction—explanation in the text. The dashed lines mark the path of the pipette delivering the eluent, the arrows indicate the directions of the eluent migration. 1—pipette delivering the eluent, 2—starting spots of the samples, 3—matrix components of higher retention than the substances of interest, 4—the zone of substances of interest (coccidiostats), 5—the low retention matrix components, 6—TLC-MS Interface head. Stationary phase: HPTLC Silica gel.

Workflow of this method is presented in Fig. 1. 2.5 g of the feed was weighed into a 50 ml polypropylene centrifuge tube. The internal standard solution and working solution were added to obtain concentrations as listed in Table 1. The sample was vigorously hand mixed. 10 ml of acetonitrile was added and again the sample was vigorously hand mixed. Crude suspension solution was directly applied on the adsorbent layer of washed and dried chromatography plate with volume of 10 μl forming starting spots of samples. Before the first step of the development of the chromatogram the starting samples spots were narrowed by methanol to eliminate radial chromatography effect of substances of interest (Fig. 1A)^26^. Then, after evaporation of solvent, the chromatograms of the samples were developed with a mixture of toluene and methanol in a volume ratio of 1:1 (v/v)^19^ to a distance of 30 mm (Fig. 1B). Based on the last research^19^, listed coccidiostats were expected to reach a distance of about 24 mm and were observed under illumination of 254 nm light and white light using the CAMAG TLC Visualizer (Fig. 1C). During the next stage of the procedure, the zones of the substances of interest were focused at the solvent front position (methanol) at distance of 26 mm. Subsequently, the substances were able to be extracted from the front position of the solvent with methanol using the TLC-MS Interface (Fig. 1D) into a 100 µl insert vial and subjected to LC–MS/MS analysis (injection volume—20 µl).

#### Procedure 2

Procedure number 2 was based on a Cronly et al*.* method^21^, which is considered as shortened version of QuEChERS (portmanteau of “quick, easy, cheap, effective, rugged, and safe”). 2.5 g of feed was weighed into a polypropylene tube. The internal standard solution and working solution were added to obtain concentrations as listed in Table 1. Then 12 ml of deionized water was added and the tube was shaken in a horizontal shaker for 15 min. Next, 25 ml acetonitrile was added and again the tube was shaken in a horizontal shaker for 15 min. 4.0 g of anhydrous MgSO_4_ and 2 g of NaCl were added and again the tube was shaken in a horizontal shaker for 15 min, then centrifuged (5100 rpm for 20 min). The clear organic layer was filtered, collected to vial and injected into the LC–MS/MS system (injection volume—5 µl).

#### Procedure 3

2.5 g of feed was weighed into tube. The internal standard solution and working solution were added to obtain concentrations as listed in Table 1. Next, 12.5 ml of acetonitrile was added and the tube was shaken for 30 min in a horizontal shaker. Then, tube was centrifuged for 10 min at 600 g. 2.5 ml of the supernatant was collected in a glass tube and evaporated to dryness under nitrogen in a water bath at 60 °C. Residues of sample were dissolved in 5 ml of acetonitrile and water (50/50, v/v) and the obtained solution was vortexed for 30 s. Then the sample was in a sonication water bath for 5 min. The extract was filtered using a 0.22 µm filter and disposable syringe directly into the 350 µl insert to the vial. The closed vial was transferred to the LC–MS/MS system (injection volume—5 µl).

#### Procedure 4

Procedure number 4 was adopted from the Dubois et al*.* method^23^, based on Solid Phase Extraction (SPE). 2.5 g of the feed was weighed into a 50 ml polypropylene tube. The internal standard solution and working solution were added to obtain concentrations as listed in Table 1. Then 5 g of anhydrous sodium sulfate was added and mixed thoroughly. Next, 7.5 ml of acetonitrile was added to the mixture. The tube was vortexed for 1 min and placed on a horizontal shaker for 60 min. Then, the tube was centrifuged at 4000*g* for 20 min at 4 °C. For the SPE cleanup: the SPE column was conditioned with 5 ml of acetonitrile; then an aliquot of the extract (≈ 6 ml) was passed through the cartridge and the eluate was collected in a 15 ml plastic conical tube; the silica cartridge was washed with another 4 ml of acetonitrile and the eluate was collected in a glass tube. The combined eluate was vortexed and 1.5 ml of this eluate was mixed with 1.5 ml of acetonitrile, filtered and transferred to a vial and analyzed by LC–MS/MS (injection volume—5 µl).

### MRM transitions

In the Table 2 MRM transitions are presented.

**Table 2:** MRM transitions.

Substance	Precursor ion (m/z)	Product ion (m/z)	Collision energy (eV)	Polarity	Cone voltage (V)
Maduramycin	939.0	877.5	50	Positive	34
Narasin	787.4	531.0	50	Positive	60
Salinomycin	773.4	531.3	32	Positive	64
Nigericin	747.5	703.4	70	Positive	60
Monensin	693.3	675.4	45	Positive	60
Lasalocid	613.6	359.3	20	Positive	48
Robenidine	334.0	110.9	50	Positive	30
Nicarbazin (DNC)	301.1	107.0	38	Negative	18

### Validation of the results

Validation was performed based on European Commission Regulation 2021/808 of March 22 2021. Chosen parameters were: linearity, recovery, repeatability, reproducibility and decision limit (CCα).

#### Linearity

Eight-point calibration curves were constructed based on the response of the corresponding ratio of the analyte peak area to the internal standard and presented in a mathematical formula. For every day of samples’ analysis calibration curves were prepared separately. Concentrations of feed extracts used to plot the calibration curve were 0.25, 0.5, 1, 1.25, 1.5, 1.75, 2 and 4 times the ML. Determination coefficient (R^2^) was calculated by least squares linear regression analysis.

#### Repeatability and reproducibility

Repeatability and reproducibility were assessed by analyzing 6 replicates of extracts from feed fortified at a constant ML level (1 ML) for three consecutive days. The experiment was performed by one researcher with different batches of reagents and solvents on different days.

The basis for determining the precision is the calculation of two parameters: RSDr and RSD_R_. RSDr is the relative standard deviation, calculated from the results generated under repeatability conditions [(Sr/x) * 100], where Sr is the standard deviation and x is the mean of the results of all 6 samples from a single analysis. The RSD_R_ is the relative standard deviation calculated from the results obtained under reproducibility conditions [(S_R_/x) * 100], during three consecutive analyses, carried out on three different days, with different batches of reagents and solvents (S_R_ is the standard deviation, x is the mean of the results).

#### Recovery

The recoveries were determined by comparing the measured concentrations to the spiked concentration.

#### Decision limit (CCα)

The decision limit (CCα) means the concentration at and above which it can be concluded with an error probability of 5% that a sample is non-compliant and the value 1 – α means statistical certainty in percentage that the permitted limit has been exceeded. It was calculated as the sum of the ML level plus 1.64 times the standard deviation of within-laboratory reproducibility at the permitted limit^20^.

### Limit of detection (LOD)

The limit of detection (LOD) is the lowest value of concentration of substance of interest, that can be reliably differentiate from sample. It was calculated for each substance of interest and procedure using the formula: LOD = 3.3σ/s, where σ is the standard deviation of the response and s is the regression line slope^27^.

## Results

### Calibration curve (linearity)

In Table 3 the mean values of the determination coefficients for the calibration curves of coccidiostats’ samples prepared by Procedures 1, 2, 3 and 4 are presented. As can be seen, the compliance of the data with the curve was very high for each drug and procedure. Very strict requirements for the coefficient of determination were adopted, because the acceptable minimum value was 0.960, which was internal criterium. All the curves met this condition, except for robenidine in Procedure 3 (0.590). Procedure 3 is probably less effective to this coccidiostat. Other explanation can be easy decomposition of robenidine under minimal influence of sunlight^28^.

**Table 3:** Average determination coefficients (R^2^) of calibration curves of coccidiostats in feed samples.

Substance	Procedure 1	Procedure 2	Procedure 3	Procedure 4
Robenidine	0.971	0.979	0.590	0.994
Nicarbazin (DNC)	0.979	0.989	0.989	0.966
Lasalocid	0.975	0.998	0.992	0.994
Salinomycin	0.984	0.998	0.996	0.999
Monensin	0.989	0.997	0.994	0.997
Narasin	0.979	0.998	0.997	0.998
Maduramycin	0.982	0.997	0.995	0.989

### Trueness (recovery)

Trueness (recovery) is the amount of analyte divided by the amount of analyte in the enriched matrix sample, expressed as percentage. According to the guidelines, correct results must fall in the range of 80–120%. Table 4 presents recoveries obtained by Procedure 1, 2, 3 and 4. Procedure 2 performed the most satisfactorily in terms of recovery (98.6–101.9%), except for nicarbazin (123.0%). Procedure 1 and 4 delivered less satisfactory results: 90.1–111.1% and 88.5% to 100.6% respectively, but all in the range described in Regulation 2021/808. Procedure 3 crossed criteria in case of robenidine (79.6%) and nicarbazin (129.7%).

**Table 4:** Recovery of coccidiostats in samples for each procedure.

Substance	Procedure 1	Procedure 2	Procedure 3	Procedure 4
Robenidine	111.0	99.8	79.6	88.5
Nicarbazin (DNC)	90.4	123.0	129.6	94.5
Lasalocid	103.2	98.6	97.0	95.5
Salinomycin	90.1	101.9	102.3	99.5
Monensin	92.5	100.8	106.5	100.6
Narasin	99.7	101.3	98.6	100.1
Maduramycin	90.9	100.0	103.3	92.2

### Within-laboratory repeatability and reproducibility (precision)

Results are presented in Table 5.

**Table 5:** Within-laboratory repeatability and reproducibility of determination of coccidiostats for each procedure.

Substance		Max level as calculated by Horwitz	Procedure 1	Procedure 2	Procedure 3	Procedure 4
Robenidine	RSDr	14.67	10.38	8.52	72.29	8.52
	RSD_R_	22.00	10.44	11.86	67.67	12.36
Nicarbazin (DNC)	RSDr	14.67	9.07	6.83	5.55	20.94
	RSD_R_	22.00	9.31	8.93	13.22	22.20
Lasalocid	RSDr	10.67	9.38	25.77	6.99	5.69
	RSD_R_	16.00	10.20	23.65	15.79	12.15
Salinomycin	RSDr	14.67	8.66	7.35	4.83	5.89
	RSD_R_	22.00	9.00	6.85	5.79	7.80
Monensin	RSDr	10.67	9.32	7.44	7.02	6.43
	RSD_R_	16.00	10.80	7.90	8.79	6.52
Narasin	RSDr	14.67	11.14	6.77	5.04	5.63
	RSD_R_	22.00	10.54	6.75	5.38	7.85
Maduramycin	RSDr	16.67	16.59	6.67	6.12	8.24
	RSD_R_	25.00	17.70	6.94	7.90	14.76

The RSDr for a single analysis must be lower than two-thirds of the calculated value of the Horwitz coefficient for a particular substance, while for the RSD_R_ for reproducibility it must be lower than the full value of the Horwitz coefficient. The values presented in the Table 5 for all tested coccidiostats mostly meet the requirements calculated individually for each substance. The exceptions are lasalocid in Procedure 2, robenidine in Procedure 3 and nicarbazin in Procedure 4. As was mentioned in previous point Procedure 3 is not effective to robenidine. High deviation of results of nicarbazin obtained by Procedure 4 could be caused by strong bonding of this substance to the SPE column. The only method that met all requirements is Procedure 1, which RSDr ranging from 8.66 to 16.59% and for RSD_R_ ranging from 9.00 to 17.70%.

### Decision limit (CCα)

The calculated CCα values are shown in the Table 6.

**Table 6:** CCα of coccidiostats for each procedure.

Substance	ML in feed (μg/kg)	Procedure 1	Procedure 2	Procedure 3	Procedure 4
Robenidine	700	833	835	1318	825
Nicarbazin (DNC)	500	568	590	640	672
Lasalocid	1250	1465	1728	1564	1487
Salinomycin	700	793	780	768	789
Monensin	1250	1454	1413	1441	1384
Narasin	700	820	778	760	790
Maduramycin	50	63	55	56	61

### Limit of detection (LOD)

Table 7 shows the results of the limits of detection for each substance of interest and procedure. The European Commission Regulation 2021/808 of 22 March 2021 does not contain detailed requirements for this parameter. However, the LOD should be below the established ML^29^ and each procedure meets this condition, except for robenidine in Procedure 3. The potential cause of which has already been clarified.

**Table 7:** Limits of detection (LOD) for each procedure.

Substance	ML in feed (μg/kg)	Procedure 1	Procedure 2	Procedure 3	Procedure 4
Robenidine	700	274	185	5911	110
Nicarbazin (DNC)	500	166	120	107	166
Lasalocid	1250	453	173	233	198
Salinomycin	700	204	67	92	58
Monensin	1250	301	136	213	118
Narasin	700	230	73	79	60
Maduramycin	50	15	6	8	10

### Duration times of each sample preparation procedure

In Table 8 duration times of each sample preparation procedure per sample are presented. Listed values refer to real-time performance, which covers extraction and purification of sample, including shaking of the tubes, centrifugation, sonication, but also weighing of salts, evaporation of solvents under nitrogen, conditioning of SPE columns, development of chromatograms or filtration. Consistently, it excludes quantitative analysis, i.e. flushing of column, injection of sample, analysis or equilibration of column. Duration time was measured for preparation of one batch of 15 samples (6 replicates of extracts, 8 samples for plotting the calibration curve and 1 standard solution for calculation of recovery). As one can see Procedure 1 is the shortest procedure among the mentioned ones, due to a very simple extraction and application of prototype of a semiautomatic device with a moving pipette for delivering the eluent to the chromatography plate. Obtained time result for Procedure 1 confirms results obtained by Klimek-Turek et al*.* with SFPE^27^. For each of the Procedures, it is possible to reduce of the duration time along with an increase in the number of samples prepared at the same time. However, there are some limitations. For Procedure 1, the maximum limit of applied samples per one chromatographic plate (10 cm × 20 cm) was 16. Exceeding this limit would lead to the necessity of application of samples onto the next plate and repeating the procedure of development of chromatogram. Similarly, 16 was limit for simultaneously extracted samples with SPE vacuum manifold in Procedure 4, but the newest manifolds have usually higher limits. Rest procedures didn’t have such limitations and their limit would be the maximum capacity of the centrifuge, which is 32 tubes per run.

**Table 8:** Times of sample preparation of each procedure per single sample.

	Procedure 1	Procedure 2	Procedure 3	Procedure 4
Time per sample	3 min	4 min 20 s	3 min 40 s	6 min

## Discussion

The main aim of the research was to optimize the SFPE for the determination of coccidiostats in order to monitor the safety of animal feeds. Currently, there are many other effective procedures for the determination of coccidiostats already established and among them three were selected for this study. They are based on main trends in sample preparation. The first procedure (here presented as Procedure 2) reported by Cronly et al*.* was developed in cooperation with the Irish The State Laboratory^21^. It represented the advantages of the original QuEChERS procedure^30^, like a high recovery and precision, and at the same time decreased costs and shortened time of analysis by eliminating the d-SPE stage. The second procedure (Procedure 3) developed by Mortier et al. at the Agricultural Research Center Ghent (CLO)^22,31^, was originally dedicated to egg samples but has been adapted for this article to feed. It represented a different trend in the sample preparation: maximum simplicity of extraction. The procedure was relatively short and easy to perform, and at the same time very effective in the determination of coccidiostats in eggs. The third procedure, designated as Procedure 4, was developed by Dubois et al*.* in the Belgian CER^23^. It was based on Solid Phase Extraction (SPE), which effective performance for biological samples has been widely proven^32–34^. The results obtained by this method were very satisfying. Moreover, SPE based procedures has a great potential to remove a large number of interferents, what results in minimal matrix effect^35^. Solvent Front Position Extraction (Procedure 1) is a method developed by employees of the Department of Physical Chemistry at the Medical University of Lublin. Like it was said before it is based on thin-layer chromatography, which is not common foundation of sample preparation of coccidiostats in biological samples, however there are exceptions^36^. It was intended to be a cheap, simple and precise method for simultaneous preparation of many samples. Recently, SFPE was presented as very effective with separation of tryptophan from human plasma^27^ and with monensin and salinomycin from samples of feed premixes^19^. What is important, effectiveness of separation of substances of interest from matrix elements could be easily observed on chromatograms (MRM chromatograms are available as Supplementary Fig. 1). In this study, SFPE was optimized for the simultaneous quantitative determination of 7 coccidiostats and validated according to Commission Regulation (EU) 2021/808 of 22 March 2021 for the first time. Obtained results for this procedure were very satisfactory, which makes it a good alternative for the most common sample preparation procedures. The main advantages of this procedure were the simplicity and briefness of extraction (3 min/sample). Additionally, using a prototype of a semiautomatic device with a moving pipette for delivering the eluent to the chromatography plate could significantly shorten the analysis time and increase the reproducibility of the results (a sample video, presenting the performance of the mentioned device, was added as Supplementary Video 1). Procedure 2 could be considered as a good compromise between the complexity of the analysis and the costs incurred to obtained results. In this procedure acetonitrile was used for extraction. Its effectiveness was enhanced by addition of extraction salts, which resulted in better recoveries so as the repeatability and the reproducibility of the assay. The only drawback of this Procedure was the much longer analysis time (4 min 20 s/sample) compared to Procedure 1. In Procedure 3 acetonitrile was also used for extraction, but without any extraction salts. In order to prevent any macromolecular contaminants getting into the apparatus, extracts were filtered right before putting into the LC device. Despite its simplicity, is was very effective for the extraction of coccidiostats in samples. The disadvantage of this procedure was potentially poor sample purification and selectivity. Procedure 4 also used acetonitrile for extraction and increased its efficiency with a single extraction salt. It was potentially more selective than Procedure 2 and 3^35^, due to the use of SPE columns, but it was very time-consuming due to the number of steps and the length of the extraction itself: 6 min/sample in comparison to 4 min 20 s/sample and 3 min 40 s/sample respectively. What’s more, the necessity of the use of SPE columns increased the cost of the procedure per sample. In addition it was less precise than the simpler Procedure 2.

## Conclusions

All presented methods are equally suitable for the effective determination of coccidiostats in feed. Obtained results are mostly very satisfactory and meet the requirements listed in European Commission Regulation 2021/808 of 22 March 2021 (linearity, repeatability, reproducibility, recovery and decision limit (CCα)). Study shows, that additional chemicals (e.g. salts) can improve statistical parameters of results, but also increase time and costs of analysis. For Solvent Front Position Extraction it was the first time it had been successfully validated for 7 coccidiostats. What’s more, SFPE can be potentially effective with rest of allowed coccidiostats, which are semduramycin, decoquinate, diclazuril and halofuginone^6^, due to their similarity to determined substances. The method can be considered as an alternative to commonly used extraction procedures for biological samples such as SPE or QuEChERS. Besides, the use of a prototype of a semiautomatic device with a moving pipette for delivering the eluent to the chromatography plate can significantly reduce the analysis time. In the close prospect, the method could be tested with other matrices, for example food of animal origin, for which the requirements for maximum coccidiostats content in the samples are more restrictive^37,38^.

## Supplementary Information

Supplementary Legends.

Supplementary Figure 1.

Supplementary Video 1.
