# Supplementary material for: Solvent Front Position Extraction and some conventional sample preparation techniques for the determination of coccidiostats in poultry feed by LC–MS/MS
Source: Sci Rep. 2022 Mar 8;12:3786. doi: 10.1038/s41598-022-07587-5 (PMC8904545; doi:10.1038/s41598-022-07587-5)
Supplement: Supplementary file 1 — Supplementary Legends. [file 41598_2022_7587_MOESM1_ESM.docx]

Legend for Supplementary Video 1

Performance of the prototype of a semiautomatic device with a moving pipette for delivering the eluent to the chromatography plate (Department of Physical Chemistry, Lublin, Poland).

In the video chromatography plate is placed horizontally, with the adsorbent layer face-up, in the horizontal Teflon chamber. The pipette is moving along one axis and delivering the mobile phase onto the surface of the chromatographic plate. Movement path of the pipette has been programmed on the personal computer. The tip of pipette is in close contiguity to the adsorbent layer (0.15 mm), without touching it. The pipette is equipped with the capillary and combined with syringe pumps (on the right).

Legend for Supplementary Figure 1

MRM chromatograms of each coccidiostat. MRM transitions are available in the manuscript in Table 2. A - MRM chromatograms for coccidiostats extracted from feed, SFPE Procedure, B - MRM chromatograms for coccidiostats extracted from feed, Procedure 2.
