# Supplementary figures and images for: Solvent Front Position Extraction and some conventional sample preparation techniques for the determination of coccidiostats in poultry feed by LC–MS/MS
Source: Sci Rep. 2022 Mar 8;12:3786. doi: 10.1038/s41598-022-07587-5 (PMC8904545; doi:10.1038/s41598-022-07587-5)

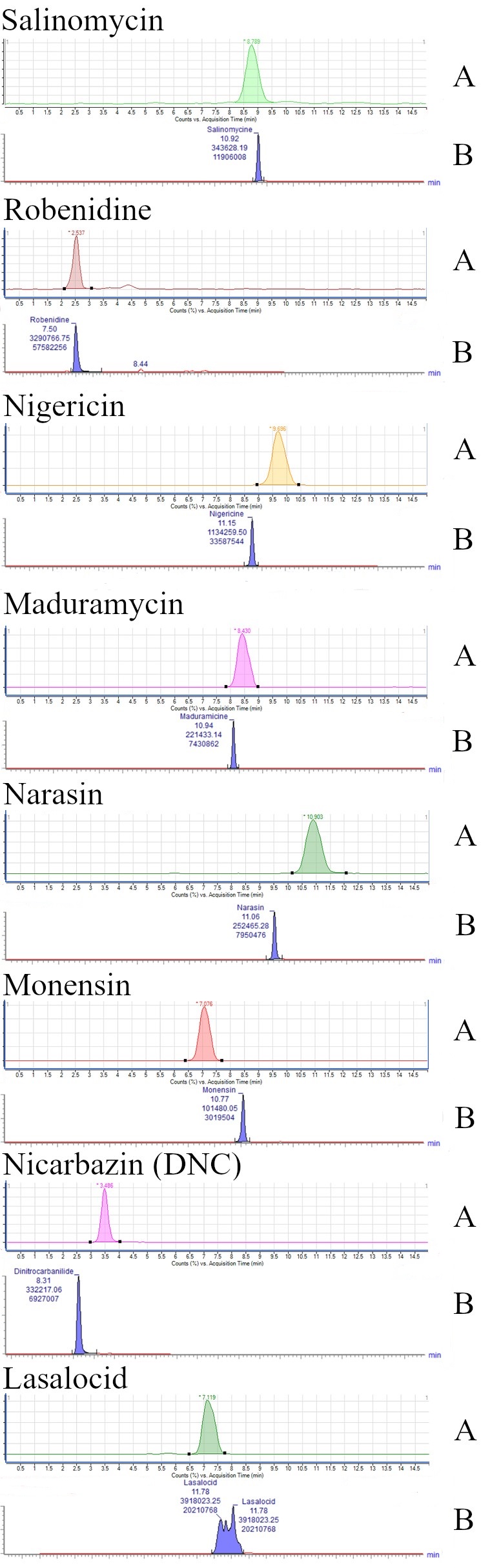

Supplement: Supplementary file 2 — Supplementary Figure 1. [file 41598_2022_7587_MOESM2_ESM.jpg]
